# Lower Somatic Mutation Levels in the *λ* Light-Chain Repertoires with Chronic HBV Infection

**DOI:** 10.1155/2021/5525369

**Published:** 2021-06-07

**Authors:** Binbin Hong, Qiaoling Liu, Qiulan Li, Lili Su, Lizhi Wang

**Affiliations:** ^1^Central Laboratory, Second Affiliated Hospital of Fujian Medical University, Quanzhou 362000, China; ^2^Traditional Chinese Medicine Department, Rehabilitation Hospital, Nan'an, Quanzhou 362300, China

## Abstract

To investigate the characteristics of the immunoglobulin light-chain repertoires with chronic HBV infection, the high-throughput sequencing and IMGT/HighV-QUEST were adapted to analyze the *κ* (IgK) and *λ* (IgL) light-chain repertoires from the inactive HBV carriers (IHB) and the healthy adults (HH). The comparative analysis revealed high similarity between the *κ* light-chain repertoires of the HBV carriers and the healthy adults. Nevertheless, the proportion of IGLV genes with ≥90% identity as the germline genes was higher in the IgL light-chain repertoire of the IHB library compared with that of HH library (74.6% *vs.* 69.1%). Besides, the frequency of amino acid mutations in the CDR1 regions was significantly lower in the IgL light-chain repertoire of the IHB library than that of the HH library (65.52% *vs.* 56.0%). These results suggested the lower somatic mutation level in the IgL repertoire of IHB library, which might indicate the biased selection of IGLV genes in the IgL repertoire with chronic HBV infection. These findings might lead to a better understanding of the characteristics of the light-chain repertoires of HBV chronically infected individuals.

## 1. Introduction

Hepatitis B virus infection is the major global health problem, despite the existence of hepatitis B vaccination [1, 2]. Most primary HBV infections are self-limiting with virus clearance and lifelong protective immunity; however, an estimated 3% to 5% of adults and up to 95% of children develop chronic HBV infection [3]. Chronic HBV infection greatly increases the risk of terminal liver disease, but current therapies could rarely achieve a cure. An extensive body of research suggested the neutralizing antibodies can not only prevent acute HBV infection but also have a possibility to modulate the development of chronic HBV infection [4, 5].

Recently, high-throughput sequencing technologies have been advanced and are improving our understanding of humoral immune responses [6–8]. Some characteristics of the antibody repertoire before and after HBV vaccination have been demonstrated using high-throughput sequencing [9–11]. B-cell receptors (BCRs) are formed by pairing of heavy chains and light chains that together specify the effector functions [12]. Unfortunately, very few studies investigate the antibody repertoire of individuals with chronic HBV infection, and even less attention has been paid to the light-chain repertoire with HBV infection. Thus, the characteristics of the light-chain repertoires with chronic HBV infection are less clear.

In this study, we analyzed the light-chain repertoires of *κ* (IgK) and *λ* (IgL) isotypes with chronic HBV infection. The characteristics of the IgK repertoires demonstrated no significant difference between the healthy and the HBV carriers. Conversely, the characteristics of the IgL repertoires showed some difference between the healthy adults and the HBV carriers, although most of the characteristics in the IgL repertoires showed no significant difference. We found that the somatic hypermutation level in the IgL repertoire of the HBV carriers was lower than that of healthy adults. Moreover, the preferred used variable (V) and joining (J) genes also showed some difference in the IgL repertoires of the healthy and the HBV carriers.

## 2. Methods and Materials

### 2.1. Samples

Totally, twenty adults were included in this study that comprised 10 inactive HBV carriers with serum HBsAg but without serum virus load increased (IHB) and 10 healthy adults (HH) who underwent a routine health check with no history of HBV infection or known major diseases. The included HBV carriers did not have active hepatitis or received antiviral treatment. The basic characteristics of the study population are summarized in Table 1.

### 2.2. Establishment of the Antibody Repertoires for Deep Sequencing

Peripheral blood mononuclear cells (PBMCs) were prepared according to the published protocols [13]. Total RNA was extracted and then reverse-transcripted to the first-strand cDNA that was used as the template to amplify the antibody sequences. The PCR amplifications were performed using a set of sense primers highly aligned to the first seven codons of the V regions and antisense primers that exhibit specificity for the last eight codons at the 3′ ends of the constant domains corresponding to the numbering schemes of the IMGT database. Two rounds of PCR amplifications were performed to produce the special isotype antibody fragments with the proper length for sequencing. Finally, the PCR amplicons were purified and subjected to high-throughput sequencing based on the Illumina Hiseq platform according to the manufacturer's protocol.

### 2.3. Sequence Processing

A series of stringent quality control criteria were applied in the data processing as published in our previous report [14]. To exclude biologically implausible sequences, IMGT/HighV-QUEST (version 1.5.1) was used for sequence annotation to identify insertion and deletion (indels) errors and classify the sequencing data into productive and unproductive sequences [15, 16]. The unproductive VJ rearrangements were eliminated from the dataset. Then, the productive sequences that contained stop codons, indel errors, and substitutions or mutations in the conserved amino acids at specified positions were excluded. Furthermore, the redundant sequences were eliminated to avoid the accumulation of one single sequence due to PCR amplification. Finally, the unique sequences with unique VJ genes, or unique CDR3 amino acid sequences defined as the unique clones, were selected for further analysis. The number of sequences after each step of processing is listed in Table 2.

### 2.4. Statistical Analyses

The clone diversity in each library was described by the Margalef index (*D*) that was defined and given by the function *D* = (*S*−1)/ln *N*, with *S* being species richness and *N* being the total number of all specimens in a sample [17]. Student's *t*-test, Pearson's chi-square test, and logistic regression analysis were used in statistical analysis. In comparative analyses, the difference significance was determined by the parameter of the effect sizes: *Cohen's d* value and the *odds ratio* (OR) were used to measure the standardized difference between two means and between two rates, respectively [18, 19]. When *p* < 0.05 (two sided), *d* ≥ 0.20, and OR ≥ 1.50 or ≤ 0.60, the difference was considered to be significant. The data analyses were performed using the R, Perl, and GraphPad Prism programs. The sequencing data have been deposited in the NCBI SRA database (PRJNA578033).

### 2.5. Ethics Statement

The blood samples were provided by the Second Affiliated Hospital of Fujian Medical University (Quanzhou, Fujian, China) with the approval of the institutional research board and the donors' consent. Procedures followed in this study were under the ethical standards of concerned institutional policies.

## 3. Results

### 3.1. The Clone Diversity

**Figure 1:**
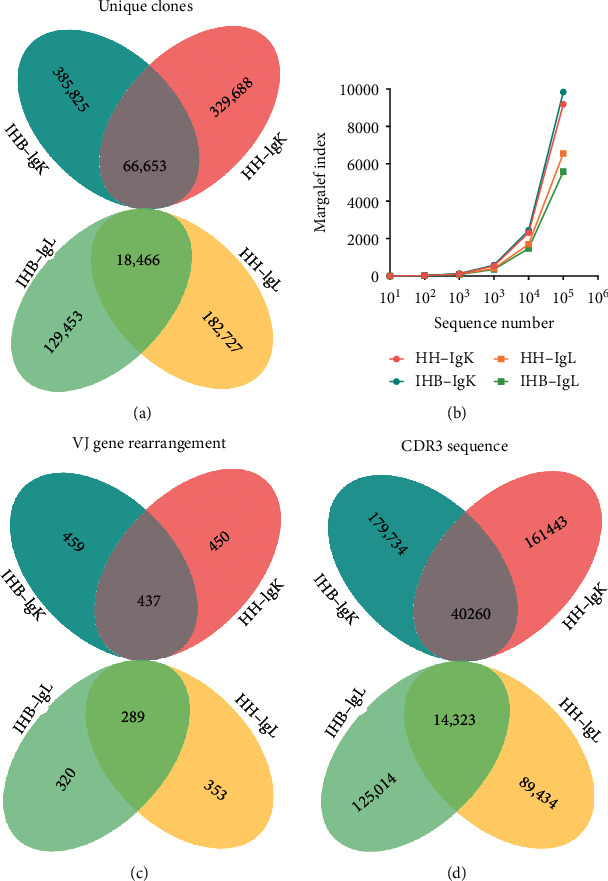
The diversity of *κ* (IgK) and *λ* (IgL) light-chain repertoires of the healthy human (HH) and inactive HBV carriers (IHB). (a) The diversity of unique clones in the IgK and IgL repertoires from the HH and IHB library. (b) The Margalef index was calculated to describe the repertoire diversity that was calculated, respectively, when 10, 100, 1000, 10000, 100000, and 1000000 sequences were randomly selected using the randomized table generated by R program. (c) The diversity of VJ gene rearrangements in the IgK and IgL repertoires from the HH and IHB library. (d) The diversity of unique CDR3 sequences in the IgK and IgL repertoires from the HH and IHB library.

In this study, we analyzed the antibody light-chain repertoires of inactive HBV carriers (IHB) and compared them with that of healthy adults (HH). Approximately 1.0 × 10^7^ PBMCs from each group were input to analyses, and totally 28,083,374 raw sequences were obtained from IgK repertoires and 25,839,997 sequences obtained from IgL repertoires after the sequencing. After a series of data cleaning procedures, a total of 385,825 unique clones were identified in the IgK repertoire of IHB library and 329,688 unique clones were found in the HH library (Figure 1(a)). Besides, 129,453 and 182,727 unique clones were found in the IgL repertoires of the IHB and HH library, respectively (Figure 1(a)). Interestingly, we found that the HH and IHB library shared 66,653 IgK clones, accounting for 17.28% in the IHB and 20.22% in the HH library, respectively (Figure 1(a)); and 18,466 clones shared between the IgL repertoires of the two libraries, constituting 14.26% of the IHB and 10.11% of the HH library (Figure 1(a)). Additionally, the Margalef index was calculated and suggested that the clone diversity demonstrated no significant difference between the HH and IHB library in both the IgK and IgL repertoires (Figure 1(b)).

Among the unique clones, 179,734 unique CDR3 sequences were found in the IgK repertoire of the IHB and 161,443 sequences were found in the HH library (Figure 1(c)); as well as 89,434 unique CDR3 sequences were found in the IgL repertoire of IHB library, and 125,014 sequences were found in the HH library (Figure 1(c)). Similarly, 40,260 convergent CDR3 sequences were found between the IgK repertoires of HH and IHB library (22.40% in IHB and 24.94% in HH); and 14,323 convergent CDR3s were found between IgL repertoires of the two libraries (16.02% in IHB and 11.46% in HH) (Figure 1(c)). Next, the VJ gene rearranged patterns were analyzed to describe the diversity of rearrangements. In IgK repertoires, 450 VJ gene rearranged patterns were found in the IHB library and 459 patterns in the HH library. We found 437 shared rearranged patterns between the two libraries that accounted for more than 95% in both the two libraries (Figure 1(d)). In IgL repertoires, 320 VJ gene rearranged patterns were found in the IHB library and 353 patterns in the HH library; and the two libraries shared 289 rearranged patterns that constituted 90.31% in the IHB and 81.87% in the HH library (Figure 1(d)). Taken together, these results demonstrated the limited diversity of unique clones in the light-chain repertoires. A small portion of unique clones and CDR3 sequences shared between the library of healthy adults and the inactive HBV carriers, but the majority of the VJ gene rearranged patterns were shared between the two libraries.

### 3.2. The Usage of VJ Genes

**Figure 2:**
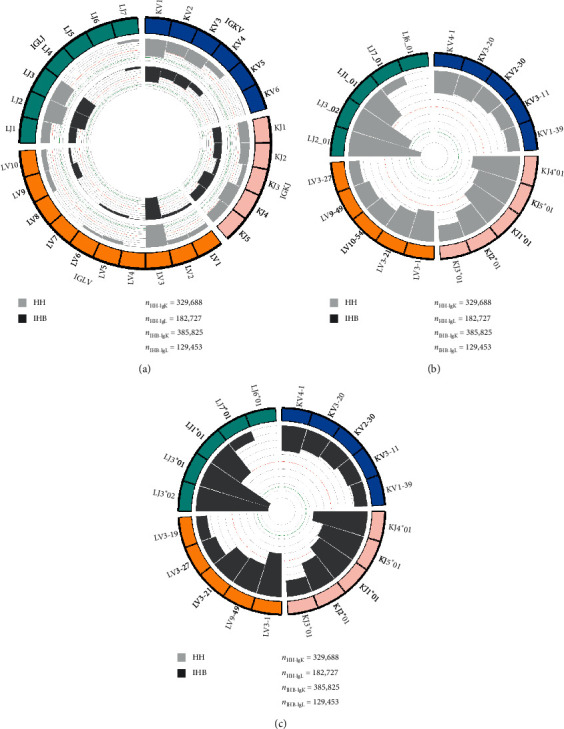
(a) The usage of VJ gene families in IgK and IgL repertoires of the HH and IHB library. The outsider arcs represent the IGKV (blue arc), IGKJ (pink arc), IGLV (orange arc), and IGLJ (cyan arc) gene families, and the histograms inside the circle represent the usage of each gene family in the IgK and IgL repertoires of HH (gray) and IHB library (dark gray). (b) The top 5 most frequently used VJ genes in the IgK and IgL repertoires of HH library. (c) The top 5 most frequently used VJ genes in the IgK and IgL repertoires of IHB library.

The IgK repertoire contains 6 IGKV gene families. IGKV1, IGKV2, IGKV3, and IGKV4 were frequently used in the two libraries, accounting for more than 98% altogether in both libraries, but IGKV5 and IGKV6 families were rarely used with a frequency less than 1.5% (Figure 2(a)). The IGKV genes usage preference was very similar between the two libraries: IGKV4-1, IGKV3-20, and IGKV2-30 were most frequently used (Figures 2(b) and 2(c), Supplementary Table 1). Besides, five IGKJ gene families were observed, all of which were frequently used. The biased used IGKJ alleles also showed no significant differences between the two libraries that were IGKJ4∗01, IGKJ5∗01, and IGKJ1∗01 (Figures 2(b) and 2(c), Supplementary Table 2). For the IgL repertoire, there are 10 IGLV gene families and 7 IGLJ gene families. The IGLV3 family was extremely biased used in both libraries accounting for 47.65% in HH and 54.58% in IHB. In both the libraries, IGLV3-1 and IGLV3-21 were frequently used. Interestingly, IGLV9-49 and IGLV10-54 were equally used in the HH library with a rate about 10%; but in the IHB library, the usage of IGLV9-49 was almost three times more than IGLV10-54 with a rate of 15.71% and 5.18%, respectively (Supplementary Table 3). For the IGLJ gene usage, IGLJ2 and IGLJ3 families were frequently used in both the IHB and HH libraries, together consisting more than 70% of all IGLJ families. The most frequently used IGLJ allele was IGLJ2∗01 in the HH library at a rate of 41.24%, but it was IGLJ3∗01 in the IHB library with a rate of 37.38% (Supplementary Table 4). Together, these results might indicate a different selection of IGLV and IGLJ genes in the IgL repertoire of IHB library, but which were not observed in the IgK repertoire.

### 3.3. The Somatic Hypermutation in V Regions

**Figure 3:**
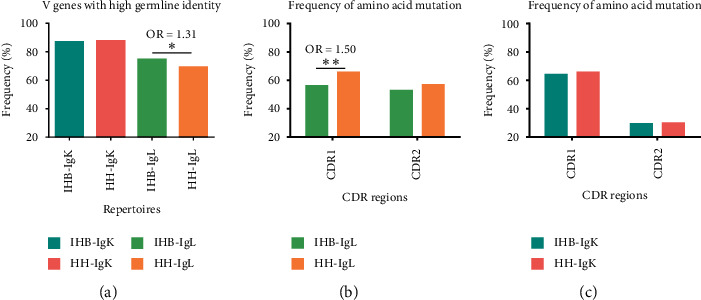
The somatic hypermutation level in the light-chain repertoires. (a) The proportion of sequences with more than 90% V gene identity as the germline genes from the IMGT database. (b) The frequencies of amino acid mutations in CDR1 and CDR2 regions of the IgL repertoires. (c) The frequencies of amino acid mutations in CDR1 and CDR2 regions of the IgK repertoires.

The somatic hypermutations (SHM) in the V gene segments were analyzed by comparing the sequencing data with the germline genes from the IMGT database. There were approximately 70% sequences in the IgL repertoires and more than 80% sequences in the IgK repertoires with ≥90% V gene identity as the germline genes (Figure 3(a)). The proportion of sequences with high V gene identity as the germline genes did not show significant difference in the IgK repertoires of HH and IHB library (HH *vs.* IHB: 87.6% *vs.* 86.9%; Figure 3(a), Supplementary Table 5). However, slight difference was found between the IgL repertoires of these two libraries (HH *vs.* IHB: 69.2% *vs.* 74.6%; *p* < 2.2E − 16, OR *=* 1.312, 95% CI*:* 1.291–1.333; Figure 3(a)), suggesting the IGLV genes in the IHB library carried less mutations.

Then, the frequency of amino acid mutations in CDR1 and CDR2 were further calculated. In IgL repertoires, it was interestingly to find that the frequency of amino acid mutations in CDR1 was lower in IHB than HH (IHB *vs.* HH: 56.0% *vs.* 65.5%; Figure 3(b)); the difference was significant since the OR value was close to the threshold level (*p* < 2.2E − 16, OR *=*1.493 ≈ 1.50, 95% CI*:* 1.472–1.515). The frequency of amino acid mutations in CDR2 was also lower in the IHB library (IHB *vs.* HH: 52.6% *vs.* 56.8%; Figure 3(b)), although the difference was not significant (*p* < 2.2E − 16, OR *=* 1.183, 95% CI*:* 1.166–1.20). In the IgK repertoires, the frequency of amino acid mutations in CDR1 and CDR2 also showed no significant difference between the two libraries (Figure 3(c), Supplementary Table 5).

### 3.4. The Characteristics of CDR3 Regions

**Figure 4:**
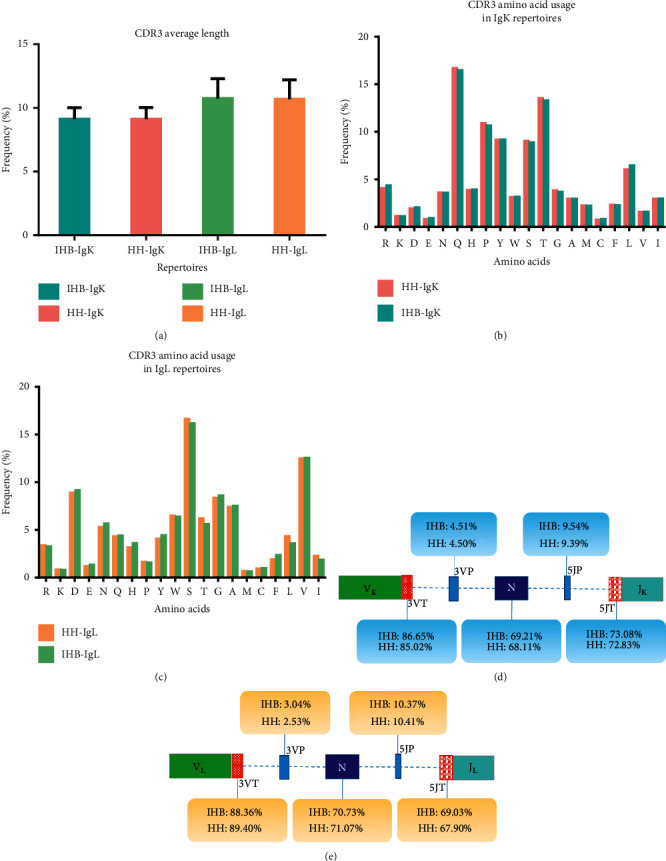
The characteristics of CDR3 regions. (a) The average length of CDR3 regions in the IgK and IgL repertoires from the HH and IHB library. (b) The usage of amino acids in the CDR3 regions of IgK repertoires from the HH and IHB library. (c) The usage of amino acids in the CDR3 regions of IgL repertoires from the HH and IHB library. (d) The occurrence of junctional modifications in the CDR3 regions of the IgK repertoires. (e) The occurrence of junctional modifications in the CDR3 regions of the IgL repertoires.

The characteristics of the CDR3 regions were analyzed, including the length of distribution, the amino acid usage, and the occurrence and mean length of junctional modifications. In our datasets, the length distribution of CDR3 ranged from 2 to 40 amino acids in light-chain repertoires. The average length of CDR3 was approximately 9 amino acids for the IgK repertoires and 11 amino acids for the IgL repertoires in the HH and IHB library (Figure 4(a)). For the amino acid usage in CDR3 regions, glutamine, threonine, and proline were the most frequently used amino acids in IgK repertoires (Figure 4(b)). But in IgL repertoires, the top frequently used amino acids were serine, valine, and aspartic acid (Figure 4(c)). Overall, the average CDR3 length and the amino acid usage showed little difference between the two libraries (data not shown). The junctional modifications are the main mechanisms contributing to the CDR3 diversity, including additions of the palindromic nucleotides (P) and the nontemplate randomized nucleotides (N), as well as the deletion of nucleotides caused by exonuclease trimming (T). The occurrences of these modifications were analyzed, but no significant difference was found between the two libraries in both IgK and IgL repertoires (Figures 4(d) and 4(e), Supplementary Table 6).

## 4. Discussion

In this study, the high-throughput sequencing method was adapted to analyze the light-chain repertoires of chronic HBV-infected individuals and healthy adults. Totally, 53,923,371 raw sequences were obtained from 20 individuals. After the analyses, we found that the characteristics in the IgK repertoires were highly similar between the healthy adults and the HBV carriers, including the somatic mutations in V regions, the average CDR3 length, and the occurrence of junctional modifications. Although most of the characteristics in the IgL repertoires were similar between the two groups of investigated population, we still found that the SHM level in V regions was lower in the IgL repertoire of inactive HBV carriers compared with that of healthy adults.

Human immunoglobulin consists of a pair of identical heavy chains and a pair of identical light chains, *κ* or *λ* alternatively. The heavy chains are the primary factor in forming the functional paratopes to realize foreign antigens [20, 21]. A number of research studies suggested that the rearrangements of the light chains aimed to silence the self-reactivity [22, 23]. Thus, the diversity of the light-chain repertoire was constrained. Despite the lack of clone diversity, the high portion of shared clones between individuals in light-chain repertoires has been reported recently [23–26]. In this study, we included 1 × 10^7^ PBMC initially and only obtained 1∼3 × 10^6^ unique clones in the light-chain repertoire from each library. The shared portion of the unique clones was approximately 10% to 20% in IgK or IgL repertoires between the two groups of investigated population. In addition, the diversity of VJ rearranged patterns was extremely limited due to the lack of the diversity (D) genes. Only hundreds of rearranged patterns were observed in both IgK and IgL repertoires, and more than 80% of the VJ gene rearrangements shared between the healthy adults and inactive HBV carriers in both IgK and IgL repertoires.

SHM is a critical mechanism to diversify the BCR repertoires [27–29]. High levels of SHM were commonly seen in the antibody repertoires after virus infection and vaccinations [10, 30–33], although the reduced mutation level was also observed in the IgG repertoire with acute dengue virus infection and the IgG repertoire after pandemic H1N1 influenza monovalent inactivated vaccine [34, 35]. In this study, we found the SHM level was lower in the IgL repertoire of HBV carriers compared with that of the healthy adults. However, the lower mutation level did not result in the decrease of the clone diversity in this study since the clone diversity showed no significant difference between the IgL repertoires of healthy adults and inactive HBV carriers. Thus, it could be inferred that the lower mutation level in the IgL repertoire of inactive HBV carriers might result from the biased selection of the germline-like IGLV genes under the influence of HBV infection.

## 5. Conclusions

In this study, we analyzed the light-chain repertoires with chronic HBV infection by the high-throughput sequencing. The repertoire characteristics were similar between the *κ* light-chain repertoires of the HBV carriers and healthy adults. Nevertheless, lower SHM levels in V regions were observed in the *λ* light-chain repertoire of the individuals with chronic HBV infection, which might indicate the biased selection of IGLV genes under the influence of chronic HBV infection. The findings in this study were relevant for understanding of the characteristics of the light-chain repertoire during chronic HBV infection.

## Figures and Tables

**Table 1:** The basic characteristics of study population.

Group	Gender (F/M)^a^	Average age^b^	HBsAg^c^	HBsAb^c^	HBeAg^c^	HBeAb ^c^	HBcAb^c^	Average HBV DNA load^d^ (IU/mL)
Inactive HBV carriers (IHB)	2/8	44.3 ± 9.27	+	—	—	+	+	<500^e^
Healthy adults (HH)	0/10	37.0 ± 9.55	—	—	—	—	—	0

^a^(F/M): female/male; ^b^ the average age had no significant difference in the three groups (Student's *t*-test, *p* > 0.005); ^c^five serological markers of HBV were tested by ELISA; ^d^HBV DNA load was tested by the real-time fluorescent quantitative PCR; ^e^the detection limit is 500 IU/mL.

**Table 2:** The number of input cells and sequencing data.

	Healthy human (HH)		Inactive HBV carriers (IHB)	
Repertoires	IgK	IgL	IgK	IgL
Total input cells	1.0 × 10^7^		1.0 × 10^7^	
Raw sequences	13,067,133	14,491,757	15,016,241	11,348,240
Productive sequences^∗^	10,782,027	10,991,517	12,484,862	8,278,375
Productive unique aa sequences	5,164,247	3,686,212	5,756,099	2,869,219
Unique clones	329,688	182,727	385,825	129,453

^∗^seqs: sequences.

## Data Availability

The sequencing data have been deposited in the NCBI SRA database (PRJNA578033).

## Supplementary Materials

Supplementary Materials Table 1 and Table 2: the usage of IGKV and IGKJ genes in the HH and IHB library. Table 3 and Table 4: the usage of IGLV and IGLJ genes in the HH and IHB library. Table 5: the comparisons of the somatic hypermutation levels between the HH and IHB library. Table 6: the comparisons of the occurrence of junctional modifications in IgK and IgL repertoires.
