# Supplementary material for: Lower Somatic Mutation Levels in the λ Light-Chain Repertoires with Chronic HBV Infection
Source: Evid Based Complement Alternat Med. 2021 Jun 7;2021:5525369. doi: 10.1155/2021/5525369 (PMC8203402; doi:10.1155/2021/5525369)
Supplement: Supplementary Materials — Table 1 and Table 2: the usage of IGKV and IGKJ genes in the HH and IHB library. Table 3 and Table 4: the usage of IGLV and IGLJ genes in the HH and IHB library. Table 5: the comparisons of the somatic hypermutation levels between the HH and IHB library. Table 6: the comparisons of the occurrence of junctional modifications in IgK and IgL repertoires. [file 5525369.f1.pdf]

Supplementary Table 1: The usage of IGKV genes in the HH and IHB library

HH-IgK	Frequency (%)	IHB-IgK	Frequency (%)
**IGKV4-1**	12.94	IGKV4-1	13.40
**IGKV3-20**	11.80	IGKV3-20	10.95
**IGKV2-30**	10.08	IGKV2-30	10.10
**IGKV3-11**	8.03	IGKV3-11	8.17
**IGKV1-39**	7.10	IGKV1-39	6.87
**IGKV2-28**	6.04	IGKV1-5	6.07
**IGKV1-5**	5.85	IGKV2-28	6.02
**IGKV1-33**	4.94	IGKV1-33	5.66
**IGKV3-15**	3.61	IGKV3-15	3.23
**IGKV1-12**	3.01	IGKV1-9	3.07
**IGKV1-16**	2.90	IGKV1-17	2.96
**IGKV1-9**	2.61	IGKV1-16	2.37
**IGKV2-24**	2.46	IGKV1-12	2.36
**IGKV2-29**	2.11	IGKV2-29	2.09
**IGKV1-17**	2.09	IGKV1-27	2.00
**IGKV1-27**	1.97	IGKV2D-29	1.76
**IGKV2D-29**	1.41	IGKV2-24	1.73
**IGKV1D-16**	1.31	IGKV1-6	1.28
**IGKV1-NL1**	1.26	IGKV3D-15	1.20
**IGKV1-6**	0.95	IGKV1-8	1.14
**IGKV1-8**	0.94	IGKV1D-16	1.07
**IGKV1D-12**	0.93	IGKV1-NL1	0.95
**IGKV6-21**	0.86	IGKV3D-20	0.92
**IGKV3D-15**	0.86	IGKV1-13	0.74
**IGKV3D-20**	0.74	IGKV1D-12	0.69
**IGKV2D-30**	0.73	IGKV6-21	0.57
**IGKV3D-11**	0.51	IGKV2D-30	0.55
**IGKV1-13**	0.44	IGKV3D-11	0.53
**IGKV6D-21**	0.40	IGKV1D-13	0.33
**IGKV2-40**	0.27	IGKV2-40	0.27
**IGKV1D-8**	0.25	IGKV1D-8	0.26
**IGKV1D-13**	0.24	IGKV6D-21	0.23
**IGKV3D-7**	0.14	IGKV3D-7	0.19
**IGKV1D-43**	0.10	IGKV1D-43	0.18
**IGKV5-2**	0.07	IGKV5-2	0.05
**IGKV2D-26**	0.03	IGKV2D-26	0.04
**IGKV1D-17**	0.01	IGKV1D-17	0.00

Supplementary Table 2: The usage of IGKJ genes in the HH and IHB library

**HH-IgK**	**Frequency (%)**	**IHB-IgK**	**Frequency (%)**
**IGKJ4*01**	26.97	IGKJ4*01	28.00
**IGKJ5*01**	22.45	IGKJ5*01	22.15
**IGKJ1*01**	21.55	IGKJ1*01	20.70
**IGKJ2*01**	14.89	IGKJ2*01	15.75
**IGKJ3*01**	9.23	IGKJ3*01	8.74
**IGKJ2*02**	3.07	IGKJ2*02	3.15
**IGKJ4*02**	1.09	IGKJ4*02	1.08
**IGKJ2*03**	0.57	IGKJ2*03	0.38
**IGKJ2*04**	0.19	IGKJ2*04	0.05

Supplementary Table 3: The usage of IGLV genes in the HH and IHB library

HH-IgL	Frequency (%)	IHB-IgL	Frequency (%)
**IGLV3-1**	17.66	IGLV3-1	22.66
**IGLV3-21**	15.01	IGLV9-49	15.70
**IGLV10-54**	11.02	IGLV3-21	14.91
**IGLV9-49**	9.54	IGLV3-27	8.08
**IGLV3-27**	6.46	IGLV3-19	5.95
**IGLV6-57**	5.05	IGLV10-54	5.18
**IGLV3-19**	4.57	IGLV6-57	4.57
**IGLV2-8**	4.08	IGLV2-8	2.99
**IGLV5-52**	3.60	IGLV5-37	2.60
**IGLV1-44**	2.82	IGLV5-52	2.39
**IGLV1-40**	2.72	IGLV1-40	2.12
**IGLV5-37**	2.46	IGLV2-14	1.97
**IGLV2-14**	2.28	IGLV1-51	1.52
**IGLV1-51**	2.00	IGLV3-9	1.36
**IGLV2-11**	1.73	IGLV1-44	1.20
**IGLV3-9**	1.57	IGLV3-25	1.15
**IGLV1-47**	1.51	IGLV1-47	1.13
**IGLV3-25**	1.41	IGLV4-3	0.86
**IGLV2-23**	0.75	IGLV2-11	0.79
**IGLV5-45**	0.66	IGLV5-45	0.64
**IGLV3-10**	0.66	IGLV2-18	0.57
**IGLV2-18**	0.65	IGLV2-23	0.52
**IGLV4-3**	0.57	IGLV3-10	0.38
**IGLV5-39**	0.49	IGLV8-61	0.31
**IGLV8-61**	0.24	IGLV4-60	0.15
**IGLV4-69**	0.22	IGLV4-69	0.05
**IGLV1-36**	0.11	IGLV1-36	0.05
**IGLV3-16**	0.07	IGLV3-12	0.05
**IGLV7-46**	0.03	IGLV3-16	0.05
**IGLV7-43**	0.03	IGLV5-39	0.05
**IGLV3-12**	0.02	IGLV7-43	0.02
**IGLV4-60**	0.00	IGLV7-46	0.02
**IGLV3-22**	0.00	IGLV3-22	0.00

Supplementary Table 4: The usage of IGLJ genes in the HH and IHB library

HH-IgL	Frequency (%)	IHB-IgL	Frequency (%)
**IGLJ2*01**	41.24	IGLJ3*02	37.38
**IGLJ3*02**	33.74	IGLJ2*01	37.33
**IGLJ1*01**	19.33	IGLJ1*01	18.97
**IGLJ7*01**	5.24	IGLJ7*01	5.43
**IGLJ6*01**	0.18	IGLJ6*01	0.35
**IGLJ5*01**	0.15	IGLJ5*01	0.32
**IGLJ4*01**	0.06	IGLJ4*01	0.20
**IGLJ5*02**	0.04	IGLJ5*02	0.03
**IGLJ7*02**	0.02	IGLJ7*02	0.01

Supplementary Table 5: The comparisons of the somatic hypermutation levels between the HH and IHB library

	**Library**		***p, OR(95%CI)*** [a]
	HH (%)	IHB (%)	IHB *vs.* HH
**IgK repertoire**			
**P ($V_{Identity}$≥90%)** [b]	87.59	86.92	<2.2E−16, 1.062(1.047,1.077)
**P(mut in CDR1)** [c]	65.47	64.01	<2.2E−16, 1.066(1.056,1.076)
**P(mut in CDR2)** [d]	29.77	29.32	<2.2E−16, 1.021(1.011,1.032)
**IgL repertoire**			
**P ($V_{Identity}$≥90%)**	69.15	74.62	<2.2E−16, 0.762(0.750,0.775)
**P(mut in CDR1)**	65.52	56.00	<2.2E−16, 1.493(1.472,1.515)
**P(mut in CDR2)**	56.79	52.63	<2.2E−16, 1.183(1.166,1.20)

a: Calculated by the logistic regression.

b: Frequency of the sequences with ≥90% V gene identity as the germline genes.

c: Frequency of the sequences with amino acid mutations in CDR1 regions.

d: Frequency of the sequences with amino acid mutations in CDR2 regions.

Supplementary Table 6: The comparisons of the occurrence of junctional modifications in IgK and IgL repertoires

Junctional modifications	Occurrence (%)				
	3VP	3VT	5JP	5JT	N
**IgK repertoire**					
**HH**	4.50	85.02	9.39	72.83	68.11
**IHB**	4.51	86.65	9.54	73.08	69.21
p*, *OR(95%CI) [a] **(HH *vs.* IHB)**	0.776, 1.003 (0.981,1.026)	<2.2E−16, 1.143 (1.128,1.159)	0.033, 1.017 (1.001,1.033)	0.017, 1.013 (1.002,1.023)	<2.2E−16, 1.053 (1.042,1.063)
**IgL repertoire**					
**HH**	2.53	89.40	10.41	67.90	71.07
**IHB**	3.04	88.36	10.37	69.03	70.73
p*, *OR(95%CI) [a] **(HH *vs.* IHB)**	<2.2E−16, 1.208 (1.157,1.261)	<2.2E−16, 0.900 (0.880,0.920)	0.719, 0.996 (0.973,1.019)	2.3E-11, 1.054 (1.038,1.070)	0.034, 0.983 (0.968,0.999)

a: Calculated by the logistic regression.
